# Obesity and Aging in the *Drosophila* Model

**DOI:** 10.3390/ijms19071896

**Published:** 2018-06-27

**Authors:** Martina Gáliková, Peter Klepsatel

**Affiliations:** 1 Department of Zoology, Stockholm University, Svante Arrhenius väg 18B, S-106 91 Stockholm, Sweden; 2 Institute of Zoology, Slovak Academy of Sciences, Dúbravská cesta 9, 845 06 Bratislava, Slovakia

**Keywords:** *Drosophila*, obesity, energy metabolism, lifespan, high-sugar diet, high-fat diet, dietary restriction, obesity paradox, AKH, IIS

## Abstract

Being overweight increases the risk of many metabolic disorders, but how it affects lifespan is not completely clear. Not all obese people become ill, and the exact mechanism that turns excessive fat storage into a health-threatening state remains unknown. *Drosophila melanogaster* has served as an excellent model for many diseases, including obesity, diabetes, and hyperglycemia-associated disorders, such as cardiomyopathy or nephropathy. Here, we review the connections between fat storage and aging in different types of fly obesity. Whereas obesity induced by high-fat or high-sugar diet is associated with hyperglycemia, cardiomyopathy, and in some cases, shortening of lifespan, there are also examples in which obesity correlates with longevity. Transgenic lines with downregulations of the insulin/insulin-like growth factor (IIS) and target of rapamycin (TOR) signaling pathways, flies reared under dietary restriction, and even certain longevity selection lines are obese, yet long-lived. The mechanisms that underlie the differential lifespans in distinct types of obesity remain to be elucidated, but fat turnover, inflammatory pathways, and dysregulations of glucose metabolism may play key roles. Altogether, *Drosophila* is an excellent model to study the physiology of adiposity in both health and disease.

## 1. Introduction

Over the last several decades, obesity has become a global epidemic. According to the statement of the World Health Organization from 2017 [1], the prevalence of obesity has nearly tripled since 1975. More than 1.9 billion adults suffer from being overweight, and of these, over 650 million are obese. Obesity increases the risk of many health problems, including diabetes, metabolic syndrome, cardiovascular diseases and cancer, and hence leads to a higher mortality [2,3,4]. However, some studies questioned the causality between adiposity and mortality (e.g., [5,6]), arguing that the increased risk of death is associated with cardiorespiratory fitness, not with body mass index (BMI) [6]. In addition, a certain proportion of obese individuals do not suffer from any health complications nor increased mortality; this condition is known as metabolically healthy obesity (MHO) [7,8,9]. The prevalence of MHO ranges between 6–75% of all obesity cases [10]. The identification of mechanisms that underlie the adiposity-related complications in some, but not all individuals, represents an important challenge in the field of obesity research.

The core metabolic pathways that regulate energy homeostasis are highly evolutionarily conserved, and the fruit fly *Drosophila melanogaster* has served as an excellent model for metabolic and diet-associated diseases (reviewed in [11,12,13,14,15,16,17,18]). For example, flies have been used to investigate the metabolic control of tissue growth, and the link between the energy metabolism and cancer [18]. *Drosophila* has also been used to model obesity induced by high-sugar (HSD) [19,20,21,22,23,24,25] and high-fat diets (HFD) [26,27,28,29]. Moreover, like humans, flies fed a sugar-rich diet suffer from hyperglycemia, insulin resistance [20,22,24], and cardiomyopathy [19]. Fly obesity can also be induced by genetic manipulations, which allows decoupling of adiposity from changes in glycemia and carbohydrate metabolism [30]. Altogether, the *Drosophila* model provides excellent tools to disentangle the effects of excessive fat storage from other obesity-related factors that might be responsible for the metabolic dysregulations and lifespan shortening in some types of obesity.

This review aims to summarize the current knowledge on the lifespan and general physiology of obese flies. In the first part, we compare the regulation of energy storage in *Drosophila* and humans; in the second part, we discuss the lifespan consequences of different types of fly obesity. Whereas some obesity-inducing diets reduce lifespan expectancy [19,28], the excessive lipid storage can be uncoupled from the lifespan shortening [23,28,31]. Moreover, many fly models of longevity are actually obese (e.g., [32,33,34,35,36]). In the last part, we therefore discuss the potential adaptive roles of obesity and insulin resistance, and the possible mechanisms whereby excessive adiposity leads to metabolic complications and lifespan shortening in some, but not all types of obesity. 

## 2. Energy Homeostasis in *Drosophila* and Humans

### 2.1. Circulating and Stored Sources of Energy

As in humans, the main circulating energy sources in *Drosophila* are sugars. However, the predominant sugar is trehalose, even though glucose is found in the fly hemolymph as well [37]. In contrast to the relatively low, but very stable glycemia in humans (0.1% glucose in the blood), insects have higher and more variable concentration of circulating sugars, with trehalose concentration in the hemolymph between 1–2% [38]. Trehalose is a non-reducing disaccharide, and therefore can be accumulated in the hemolymph at high levels without any detrimental effects [38]. Conditions that increase trehalose levels—such as a high-sugar diet—also increase circulating glucose [20]. Glucose can at least partially compensate for the lack of trehalose, as mutants deficient in trehalose production survive until the late pupal stage [39]. Before being utilized by cells, trehalose needs to be converted into glucose by trehalase [40,41,42]. Many studies therefore report just the total glycemia (e.g., [26,37,43,44]), without differentiating between the circulating sugars.

In contrast to the tight regulation of glycemia in humans, flies tolerate considerable fluctuations in the circulating sugars, from 50% reduction (e.g., [37,43]) to more than 50% increase (e.g., [33]). In humans, hyperglycemia leads to a damage of vascular endothelial cells, causing impairment of blood vessels, cardiovascular diseases, kidney failure, and blindness (reviewed e.g., in [45]). As *Drosophila* has an open circulatory system, damage to the vascular cells is not an issue. Altogether, hyperglycemia does not seem to be detrimental in this model system. Moreover, several manipulations that extend lifespan are, in the fruit flies, accompanied by increased levels of circulating sugars [21,33].

**Figure 1:**
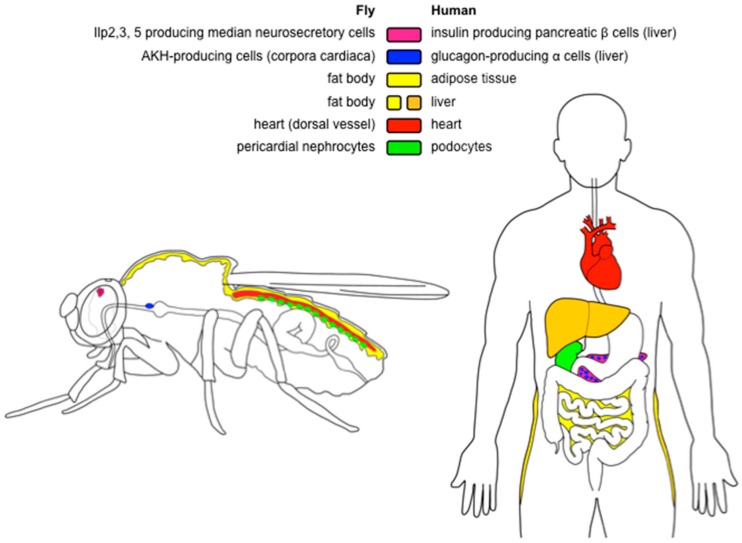
The organs that regulate energy homeostasis and may link obesity to lifespan. The tissues that share similar functions in *Drosophila* and humans are depicted in the same colors. The median neurosecretory cells of the fly brain produce Insulin-like peptides 2, 3, and 5 (Ilp2, 3, and 5), which act similarly to human insulin produced in the pancreatic beta cells. The *Drosophila* analog of human glucagon—adipokinetic hormone—is produced in the corpora cardiaca, whereas human glucagon is produced in the alpha cells of the pancreas. In the fly, fat and glycogen are stored in the fat body, which is an organ fulfilling the functions of the human adipose tissue and liver. The dorsal vessel (fly heart) is a linear tube that pumps hemolymph into the open circulatory system, and is considered as a functional counterpart of the human heart. Pericardial nephrocytes are cells that filter fly hemolymph, and share morphological and functional features with podocytes, cells of the kidney glomerulus. See the text for further details.

**Figure 2:**
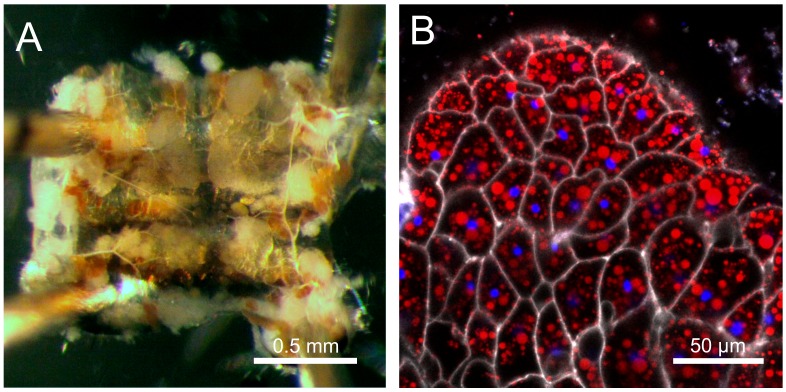
The fat body of *Drosophila*. (**A**) Bright-field image of a subcuticular fat body (white tissue) attached to the abdominal cuticle. (**B**) Confocal microscope image of the fat body. Cell membranes in white (CellMask Deep Red), lipid droplets in red (BODIPY 493/503) and DNA in blue (Hoechst 33342).

Like mammals, *Drosophila* stores excess chemical energy in the form of glycogen and lipids. Although glycogen accumulates also in muscles [46], the main storage organ for both carbohydrates and lipids is the fat body [46,47]—an organ analogous to the human adipose tissue, but also performing the functions of the liver [47] (Figure 1). The fat body consists of polyploid, sometimes multinucleate cells, which store lipids in specialized organelles called lipid droplets (Figure 2). Lipid droplets are conserved from yeast to humans, and their size and numbers per cell are highly variable [48,49]. However, apart from the increased number and volume of lipid droplets, very little is known about the obesity-related changes of the fat body. 

### 2.2. Regulation of the Energy Balance by the Insulin-Like and Glucagon-Like Pathways

In mammals, the balance between circulating and stored energy sources is regulated by the antagonistic action of glucagon and insulin pathways. Whereas insulin triggers conversion of circulating sugars into the energy reserves, glucagon increases glycemia by promoting catabolism of fat and glycogen [50,51]. Flies have functional homologs of both insulin [32,33,52] and glucagon-like signaling [37,43,53], but not all the functions are analogous. For example, in the fly, glycogen mobilization is independent of the glucagon-like signaling [54].

*Drosophila* has eight insulin-like peptides (Ilp1–8) and a single insulin-like receptor, InR. As this pathway fulfills functions of both insulin and IGF (insulin-like growth factor) signaling, it is often abbreviated as IIS (insulin/insulin-like growth factor signaling) [33,55,56]. Ilp2, 3, and 5 are of special importance from the perspective of glycemia control. These hormones are produced in the median neurosecretory cells of the brain (Figure 1), and act similarly to human insulin produced in the pancreatic beta cells [32,52]. Mechanisms that regulate activity of these cells during development differ from those that act during adulthood [57]. For example, median neurosecretory cells of the larvae are not able to sense circulating sugars, and their activity is, therefore, regulated indirectly via the glucagon-like adipokinetic hormone (AKH) [58], and several other hormones [59,60]. By contrast, median neurosecretory cells of adults sense circulating glucose directly, via a mechanism similar to mammalian pancreatic β cells [61]. 

Fat body-specific manipulations of the InR indicate that analogously to human insulin, the fly IIS is a positive regulator of fat storage. Overexpression of a constitutively active isoform of InR increases fat content, while dominant negative isoform of InR reduces it [62]. On the other hand, several studies revealed that a global decrease of the IIS leads to obesity, as seen, for example, in the *Ilp2-3*,*5* triple mutants [33], and in the flies with ablation of the insulin-producing median neurosecretory cells [32]. Similarly, genetic manipulations of the regulators of IIS from the IGF-binding family of proteins suggest that IIS is a negative regulator of fat reserves [63,64,65].

The role of the IIS in glycogen storage of flies seems to be analogous to the function of mammalian insulin. A recent study by Yamada et al. [47] showed that similarly to insulin in mammals [50], the fly IIS positively regulates glycogen synthesis. Nonetheless, ablation of the insulin-producing cells in the brain [32] or *Ilp2-3*,*5* deficiency [33] lead to increased glycogen storage, suggesting that *Drosophila* has an additional IIS-independent factor that promotes glycogen synthesis.

In the fly, hyperglycemic functions are governed by the glucagon-like adipokinetic hormone AKH, which is produced in the endocrine organ called corpora cardiaca (Figure 1) [37,43,53]. Larval corpora cardiaca are localized in the ring gland [37,53]. The ring gland dissociates during metamorphosis, and the corpora cardiaca migrate towards the thorax, where it attaches to the esophagus, and sends axon-like projections toward the brain and crop [53]. AKH regulates catabolism of lipids analogously to mammalian glucagon. Thus, *Akh* mutants are obese [43,54], whereas *Akh* overexpression results in a lean phenotype [53,54]. However, in contrast to glucagon, AKH does not induce catabolism of glycogen [43,54] and glycogen levels rise upon *Akh* overexpression [54]. This is particularly intriguing, as AKH is a hyperglycemic hormone [37,43,53], and glycogen has been considered as the main source of trehalose [38]. Thus, the regulation of circulating and stored energy sources is more complicated, and we are still far from a complete understanding of the pathways that govern energy homeostasis in the *Drosophila* model. 

## 3. Lifespan in Different Types of Fly Obesity

There are several methods to measure the fat content of a fly, but the most common approach is based on a colorimetric measurement of glycerides [66]. The fat levels are either expressed as absolute raw values, as fat values normalized to body weight, or as fat values normalized to the protein content. In contrast to the clear BMI-based definition of overweight and obesity in humans, obesity in flies is not exactly defined, and the term is used as an equivalent for increased fat storage.

### 3.1. High Sugar Diet (HSD)-Induced Obesity

Sugar-rich substrates represent a natural food source of fruit flies [67]. However, a high sugar diet (HSD) produces obesity and hyperglycemia [20]. HSD typically refers to a fly diet containing around 30% sugar, which accounts for an approximate increase in the fat content by 50–150% [19,20,21,23,68,69]. The HSD diet is prepared by adding excessive sucrose [19,20,22,23,31], glucose [21,70], or fructose [70] to the standard fly medium. HSD decreases several aspects of fly fitness; for example, delays larval development [20,23], reduces fecundity [71,72,73], and increases the age-independent mortality [23,72]. However, the overall effect on aging is not clear. Whereas some studies reported a lifespan-shortening effect of HSD [69,72], Galenza and colleagues [21] revealed that despite causing obesity and hyperglycemia, HSD leads to a remarkable lifespan extension by 31%. Interestingly, their study suggests an increase in the early mortality of flies reared on HSD, despite an overall lifespan extension in the majority of the flies [21]. The same phenomenon was described in the work of Tânia Reis [23], who showed that the mortality of the HSD flies has a bimodal distribution, although the mean lifespan upon HSD treatment is mildly increased. 

HSD leads to an impairment of the IIS [20,21,22], although the mechanism is not entirely clear, and may depend on the developmental stage. A study by Pasco et al. [22] showed that in larvae, HSD increases expression of *Ilp2*, *3*, and *5*. However, Ilps cannot activate the InR receptor due to the increased levels of the Ilp-binding protein, lipocalin neural Lazarillo. Thus, HSD-fed larvae are insulin resistant, and their peripheral IIS is attenuated [22]. Musselman et al. [20] showed that the HSD-fed larvae are resistant even to recombinant insulin. The situation is likely different in adults, where HSD decreases the IIS independently of insulin resistance, causing decreased expression of *Ilp2*, *3*, and *5* [24], whilst the periphery remains insulin sensitive [21].

Altogether, HSD reduces the peripheral IIS, either via decreased production of insulin, or via insulin resistance. Interestingly, the survival curves of the *Ilp2-3*,*5* triple mutants [33] are reminiscent of the survival curves of flies fed HSD, that were described by Tânia Reis [23]. Moreover, the *Ilp2-3*,*5* triple mutants [33] also recapitulate other HSD-associated impairments of development and reproduction [20,22,33,72], suggesting that the reduced IIS is responsible for many disorders linked to HSD. Nevertheless, the reduced IIS cannot account for the HSD-induced lifespan shortening detected in some studies [69,72], as attenuation of IIS is typically coupled with lifespan extension [32,33]. 

The lifespan extension reported for some obese and hyperglycemic HSD-fed flies [21,23] suggests that the adiposity itself is probably not the sole cause of the reduced lifespan in other HSD-feeding experiments [19,72]. Work of Na and colleagues [31] documented that HSD leads to dysfunction of pericardial nephrocytes, cells that filtrate fly hemolymph and act analogously to human podocytes in the kidney (Figure 1). This damage is associated with increased hexosamine flux and the Polycomb gene complex activity. Interestingly, pharmacological inhibition of the hexosamine pathway extends the lifespan on HSD—seemingly beyond the lifespan of controls kept on the standard food—while reducing fat levels only partially [31]. Thus, the HSD-induced damage of nephrocytes—rather than the adiposity itself—is responsible for the lifespan-shortening effect of this diet. In addition to causing dysfunctions of nephrocytes [31], HSD affects other processes that might contribute to the reduction of fly fitness. For example, a short-term exposure to HSD decreases lifespan via a transient inhibition of the IIS-repressed transcriptional factor Foxo (Forkhead box, sub-group O), which causes a long-lasting reprogramming of the signaling in the fat body [74]. Sugar overload also directly affects numerous fat body-unrelated processes; for example, it increases endoplasmic reticulum stress [68], decreases immunity [68], disrupts gut homeostasis, and reduces commensal bacteria [75]. Like in humans, HSD causes in flies heart disorders such as fibrillations, asystolic periods, and arrhythmias, leading to progressive heart failure [19]. The detrimental effect of HSD on the heart is mediated solely by the hexosamine flux, and the cardiac-specific reduction of this pathway fully protects the heart from the HSD-induced pathologies [19]. In addition, the recent RNA-seq analyses of the HSD-induced transcriptional changes may provide further useful hints on the metabolic changes elicited by the sugar overfeeding [76]. 

Flies fed on HSD (e.g., [19,20,21,23,68,69,70]) do not have access to any additional water source to compensate for the increased osmolarity of their diet. Part of the pathologies associated with HSD may, therefore, result from the hyperosmolarity or hypovolemia. A study by Rovenko et al. [70], showed that the HSD-fed flies have indeed reduced body water content, further supporting this hypothesis. 

In conclusion, HSD may affect the fly physiology independently of the obese phenotype, for example, via direct damage of the heart [19], nephrocytes [31], or gut [75], via reprogramming of signaling pathways in the fat body [74], or via reduction of the body fluids [70]. The mechanism that circumvents these complications in the glucose-based HSD [21] remains to be elucidated, nevertheless, it might contribute to a better understanding of the MHO in humans as well.

### 3.2. Dietary Restriction and the Paradox of a Carbohydrate-Rich Diet 

Lifespan extension by dietary restriction (DR) is a phenomenon conserved from yeast to humans. There are several methods to apply DR: intermittent fasting, restriction of certain nutrients, or food dilution [77,78,79]. Diet dilution is a frequently used method in various animal models, yet several studies showed that this treatment leads to increased, compensatory feeding. This was explained by the protein leverage hypothesis [80], which states that the reduction of proteins increases appetite. Thus, when the DR food is provided ad libitum, animals consume relatively higher proportion of carbohydrates and calories, which leads to obesity [80,81]. This mechanism seems to be evolutionarily conserved, with evidence for it found from fruit flies [82] to humans [80,83,84]. In the *Drosophila* field, the DR regime typically refers to the protein restriction. Restriction of dietary yeast, the main source of proteins in the fly diet, increases both lifespan and fat reserves [35,36,82,85]. Geometric framework studies in *Drosophila* showed that ad libitum feeding on high carbohydrate/low protein diet extends lifespan, whereas the caloric restriction itself does not [71,81,86,87]. Similar data were obtained in mice [88]. The question then arises: what is the mechanism whereby the high carbohydrate/low protein diet extends lifespan? DR reduces IIS activity [89], but the lifespan extension in flies is mediated by the target of rapamycin (TOR) signaling [35]. Interestingly, pharmacological inhibition of the TOR pathway by rapamycin not only extends lifespan [34], but also increases fat reserves [34,35]. 

Thus, both DR and genetic inhibition of the nutrient-sensing pathways extend lifespan and increase fat storage [32,34,35,36,85]. How exactly the obese phenotype contributes to the longevity is unknown. However, two studies [90,91] revealed that the DR-mediated lifespan extension is associated with a beneficial effect of increased fat turnover. This process is mediated by the lipolytic AKH hormone [91] and requires the peripheral circadian clock in the fat body [90]. It remains to be investigated whether a similar increase in the lipid metabolism accompanies obesity in other long-lived models, and whether an experimental increase in the fat turnover would rescue longevity of the short-lived obese flies. 

### 3.3. The HFD-Induced Obesity

Natural food sources, as well as the standard culture media for *Drosophila*, are relatively poor in fat content. Nevertheless, flies can feed and survive on artificial experimental diets with added fat. The HFD for *Drosophila* typically contains 20% [92] to 30% [26,29,93,94] coconut oil, or 15% lard [28,95]. HFD leads to obesity [26,28,29,92], hyperglycemia [20,26,28], reduced cardiac contractility, ectopic accumulation of fat in the heart, and to other pathologies reminiscent of the diabetic cardiomyopathy [26]. HFD is also associated with lifespan reduction [28,29]. 

HFD increases TGF-β signaling, which is responsible for the development of insulin resistance [92]. Experimental reduction of TGF-β is sufficient to ameliorate both obese and hyperglycemic phenotype [92], but whether this treatment restores lifespan is not known. Interestingly, genetically enhanced lipolysis targeted to the heart is sufficient to prevent the HFD-triggered cardiac dysfunctions [26,93], suggesting that HSD causes cardiomyopathy in a tissue-autonomous manner via ectopic lipid storage. Ectopic fat accumulation appears to be a common mechanism behind the HFD-induced cardiac dysfunction also in mammals—a heart-specific increase in lipolysis improves the heart functions in mice as well [96,97,98].

Similarly to human obesity [99,100], HFD leads, in flies, to an overactivation of immune responses [28]. A macrophage-specific knockdown of the immune response restores insulin sensitivity and ameliorates the lifespan shortening, but nevertheless, does not ameliorate the HFD-induced obesity [28]. Whether and how the inflammatory pathways affect heart functions is, nevertheless, not known. In conclusion, it seems that—at least in the HFD model—lifespan is not shortened by the adiposity, but by the inflammatory pathways.

### 3.4. Obesity and Longevity in the Flies with Abrogated Reproduction

Reduced reproduction is associated with increased fat accumulation and longevity in multiple organisms (reviewed in [101]). Conversely, numerous treatments that result in longevity and excessive fat storage decrease fertility. In *Drosophila*, these examples include DR and inhibition of the nutrient-sensing pathways [32,33,34,102]. However, there are also cases where reduced reproduction correlates with obesity, but not with lifespan extension. *Drosophila* females homozygotic in the naturally-occurring allele *female sterile (2) adipose* are sterile and obese, yet not long-lived [103]. In addition, lifespan-extension associated with inhibition of breeding sometimes correlates with reduced fat reserves. For example, virgin *Drosophila* female flies have lowered starvation resistance, indicating that they are leaner than their reproducing siblings [104]. 

The interactions between the fat storage and lifespan might be partially explained by the trade-offs between energy allocation to egg production and fat storage (reviewed in [105]). Importantly, in addition to the direct costs of reproduction, fly gonads may regulate metabolism and lifespan by modulating the main signaling pathways, such as the IIS and steroid signaling. As a case in point, elimination of the germline cells in the ovaries or testis extends lifespan, and this effect is coupled with attenuated IIS [106]. 

Fly gonads also produce ecdysteroids [107,108], the only steroid hormones in *Drosophila*, which could mediate the trade-offs between reproduction and lifespan [109]. Although the experimental evidence in females is equivocal, reduced ecdysteroid levels or mild RNAi against the Ecdysone receptor (*EcR)* extends lifespan in males [110,111]. Interestingly, the functions of ecdysteroids in adult flies are reminiscent of the roles of human sex steroid hormones estrogen and testosterone. Like sex steroids, ecdysteroids regulate maturation (metamorphosis) [112] and reproduction [113,114,115]. In larvae, *EcR* acts as a negative regulator of fat accumulation [116], and it is possible that the same role is conserved in adults as well. In that case, *Drosophila* could be an appropriate model to study the obesity associated with reduced levels of sex steroids, a condition which occurs in humans after gonadectomy, menopause, or during aging [117,118,119].

### 3.5. Genetic Links between Fat Storage and Lifespan 

The typical genetic interventions that result in a lifespan extension—such as attenuation of the IIS and TOR signaling—also result in obesity [32,33,34,35,36]. Similarly, the long-lived *Methuselah (Mth)* mutants have increased starvation resistance [120], indicating that their fat stores are increased. In addition, several selection experiments revealed existence of a genetic basis for the positive correlation between obesity and lifespan. For example, some *Drosophila* lines selected for longevity have increased resistance to starvation, suggesting higher energy reserves [121,122]. Conversely, selection for increased starvation resistance resulted in increased fat storage [123,124], suggesting that longevity and obesity are determined by the same genetic variants. Nevertheless, there are also longevity selection lines without increased resistance to starvation [125], as well as fly lines with reduced body fat [126]. Thus, the longevity can evolve in various ways, which may or may not involve an increase in the body fat.

Altogether, several selection experiments revealed the existence of a genetically based variation in the fat metabolism and lifespan, suggesting that the fat storage and longevity share a common genetic architecture [121,122,123,124]. The recent advancement of sequencing technologies, together with the genome-wide association studies on the inbred lines from the *Drosophila* Genetic Reference Panel, provide additional tools for identification of genes underlying the variation in fat storage and lifespan in wild-type flies [127,128,129,130]. Further studies of these adiposity-related alleles thus hold new promises for a better understanding of the fitness and lifespan in naturally obese flies.

### 3.6. Lifespan in Other Genetic Models of Obesity 

Fly genetics provides numerous tools to induce obesity, such as manipulations of genes associated with adiposity in mice or humans, as well as mis-expressions of metabolic genes identified by the genome-wide RNAi screens in *Drosophila* [30,131,132]. These genetic manipulations allow for the analysis of the consequences of obesity without altering diet or the nutrient-sensing pathways. For example, severe obesity can be triggered via inhibition of either lipolytic pathway, one acting via the lipase Brummer, the other via the AKH hormone signaling [133]. Mutation of *brummer (bmm)* results in doubling of the fat, but only in a mild reduction of lifespan [134]. Similarly, the fat content of the *Akh* mutants is increased by around 75–100% [43,54], however, the lifespan is reduced only moderately (MG and PK; unpublished data [135]). On the other hand, increased lipolysis caused by *Akh* overexpression or by activation of the AKH secretion is associated with longevity [91,136]. Reduction of fat storage is, nevertheless, not sufficient for lifespan extension, as seen, for example, in the lean but short-lived mutants deficient for the *α-Esterase-7* [137].

In contrast to HSD- or HFD-induced obesity, genetic interventions enable distinguishing the effects of adiposity from the consequences of hyperglycemia. For example, obesity associated with euglycemia can be triggered by manipulations of calcium signaling via *Stim* RNAi [30], whereas obesity coupled with hypoglycemia can be induced by the *Akh* mutations [43]. 

Altogether, as summarized in Table 1, some types of obesity are associated with lifespan shortening, whereas others with longevity. 

## 4. Pathways and Tissues Linking Lipid Metabolism and Lifespan

Energy homeostasis of *Drosophila* is controlled by a complex neuroendocrine system with extensive interorgan communications (reviewed e.g., in [139,140,141,142,143]). Here, we focus only on the most prominent tissues and pathways that could link obesity and aging.

### 4.1. The IIS and AKH Endocrine Systems

Human obesity is often associated with profound changes in the production of adipose tissue-derived factors, such as leptin, adiponectin, and tumor-necrosis factor alpha (TNF-α). These dysregulations lead to reduced glucose uptake, increased glycemia, and development of insulin resistance (reviewed in [144,145])., Hyperglycemia-associated glucotoxicity damages, among other tissues, also pancreatic β cells, which further exacerbates the diabetic phenotype [145]. Similarly, many types of *Drosophila* obesity correlate with changes in signaling via fat body-produced hormones. For example, both HSD and HFD increase expression of the fly homolog of leptin, *unpaired2 (upd2)* [59]. Interestingly, the typical models of fly obesity (flies reared on DR, HSD, or HFD) also have reduced insulin-like signaling [21,89,92,146]. The mechanism of the IIS attenuation seems to be diet-specific. Whereas DR selectively decreases production of Ilp5 [89,146], HSD in adult flies reduces expression of all three brain-produced Ilps [24], while the periphery remains insulin-sensitive [21]. By contrast, the HFD-induced changes in the brain-produced Ilps are less clear [26,92]. Nevertheless, HFD leads to reduced peripheral IIS, and to insulin resistance [92]. Altogether, the diet-induced obesity of flies appears to be accompanied by reduced IIS, reminiscent of the diabetic phenotype of many obese patients. However, in contrast to humans, the attenuation of insulin signaling does not endanger health of the flies, but—on the contrary—extends their lifespan [32].

Diabetes and hyperglycemia in humans are associated with increased glucagon production, which further worsens the diabetic phenotype [145]. Interestingly, HSD in flies leads to increased signaling via the analogous AKH hormone [147]. Nevertheless—in contrast to humans—overactivation of the AKH signaling extends lifespan [91,136]. 

In summary, the diet-induced dysregulation of the IIS and AKH signaling in obese flies resembles the dysregulations of the corresponding pathways in obese humans. However, the health- and lifespan-associated consequences of these dysregulations differ tremendously between flies and humans.

### 4.2. The Fat Body

Similar to the human adipose tissue [144], the insect fat body is not only a deposit of neutral fat [148], but also an important endocrine and immune organ [82,139,141,148]. In addition, the fat body has a key role in the sensing of proteins and carbohydrates, and in the regulation of the hypothalamus-like centers in the brain according to these dietary cues [82]. These endocrine functions are mediated by several hormones secreted from the fat body, including the leptin analog Upd2, which signals the nutritional state by acting on the GABAeric neurons that regulate secretion of Ilp2 [59]. There are several other factors derived from the fat body that regulate the insulin-producing cells in the brain, including the peptide CCHamide-2 (CHHa2) with important developmental roles [60,149], and the satiety signal encoded by the *female-specific independent of transformer (fit)* [150]. 

The fat body negatively regulates the production of Ilps in the mid-brain via Ilp6 [138]. The fat body-specific overexpression of *Ilp6* extends lifespan and fat storage [138], thus mimicking the ablation of the insulin-producing cells in the brain [32]. In addition to the regulation of insulin production, the fat body inhibits the IIS in the periphery by secreting Impl2, an Ilp2-binding protein from the IGF-binding protein family [64]. The fat body remotely regulates additional pathways and processes; for example, slows down the digestion via secreting the Activin-like ligand Dawdle, a repressor of digestive enzymes [151].

During larval development, the fat body has an important role in coupling nutrient availability to growth. The nutrient sensing occurs via several pathways, including the amino-acid transporter Slimfast, the TOR pathway [141], and the pathway activated by the systemically circulating lipoprotein-associated form of the signaling protein Hedgehog (Hh) [152]. The circulating Hh is produced by the midgut. The expression of *hh* increases upon starvation, when the Hh pathway promotes mobilization of lipid reserves in the fat body [152]. Interestingly, the Hh signaling was identified as the top-scoring anti-obesity pathway in a genome-wide obesity screen in adult *Drosophila* [132]. Hh plays a crucial role in fat metabolism also in mammals [132], and is considered an antagonist of aging and aging-associated diseases [153].

Many lifespan-extending manipulations, including those concerning the IIS and TOR pathways, act via the fat body, and they are frequently coupled with obesity. For example, the fat body-directed overexpression of *foxo*, a transcriptional factor repressed by the IIS, extends lifespan and increases lipid accumulation [154]. The nutrient-sensing TOR pathway regulates lifespan via the fat body as well [155]. Similarly, the fat body-specific inhibition of steroid signaling via *EcR* RNAi is sufficient for the lifespan extension [111]. 

### 4.3. The Heart

Several studies have shown the importance of the heart in a remote regulation of fat reserves. For example, a heart-specific signaling via Skuld—a subunit of the mediator transcriptional complex—remotely regulates the fat storage in flies [156]. Similarly, the mammalian homolog of Skuld, MED13, remotely regulates fat storage in mice [157]. The fly heart produces a substantial proportion of the apoB-lipoproteins—the essential lipid carriers [158]. Under HFD, the apolipoproteins derived from cardiomyocytes, and not those produced by the fat body, are the predominate regulators of lipid metabolism [158]. 

As described in the part on the diet-induced obesity (Section 3.1 and Section 3.3), the HSD- or HFD-induced cardiomyopathies [19,26,93] may contribute to the lowered fitness in these types of obesity. The heart damage seems to occur in a tissue-autonomous manner, either via the increased hexosamine flux (in the case of HSD) [19], or via the overactivation of the TOR pathway and ectopic fat accumulation (in the case of HFD) [93]. However, it is unknown whether the heart accumulates ectopic fat only under the HFD [93] and HSD [19] regimes, or also in other types of obesity. Similarly enigmatic is the mechanism that putatively prevents heart dysfunctions in the long-lived obese flies. 

### 4.4. The Immune Cells

Human obesity is coupled with a chronic low-grade inflammation and activation of the immune response, which leads to insulin resistance and metabolic syndrome [99,100]. The precise mechanism behind the obesity-associated inflammation is not entirely clear. The immune response involves an increase in macrophage numbers, infiltration of the adipose tissue and other organs with macrophages, and enhanced production of proinflammatory cytokines TNF-α, interleukin 6 (IL-6), leptin, visfatin, resistin, and others [100,159]. These cytokines trigger chronic inflammation, which subsequently causes insulin resistance, hyperglycemia, and other metabolic complications [100]. In *Drosophila*, feeding on HFD leads to an immune response as well, namely, to the enhanced production of the macrophage-derived cytokine Upd3, which is responsible for the subsequent development of hyperglycemia and insulin resistance [28]. The elegant study of Woodcock and colleagues [28] showed that HFD shortens lifespan solely via the Upd3-dependent inflammatory response, and not via the increase in fat storage. It remains to be investigated whether HFD activates, in flies, production of other cytokines, and whether all types of fly obesity cause activation of the immune response. Altogether, it is possible that the activation of an immune response is a common feature triggered by adiposity. However, how is this process initiated under HFD, and avoided in the long-lived obese flies, remains to be investigated. 

### 4.5. The Pericardial Nephrocytes 

In humans, inflammation plays a central role in the development of diabetic nephropathy—end-stage kidney disease associated with diabetes [160,161]. Although accompanied by changes in several cell types in the kidney, diabetic nephropathy is caused mainly by the injury of podocytes, specialized cells that encircle capillaries of the glomerulus [162,163]. Nephrocytes—specialized cells that filter hemolymph—are the functional homologs of mammalian podocytes in the fly [164,165]. Analogous to the diabetic conditions in humans, HSD in flies leads to damage of the pericardial nephrocytes, which is a condition reminiscent of the diabetic nephropathy [31]. The podocyte injury is induced by increased hexosamine flux and enhanced activity of the Polycomb gene complex. Interestingly, inhibition of this pathway is sufficient to protect from the lifespan-shortening effect of HSD [31]. 

Altogether, the immune response and the increase in the hexosamine flux are responsible for the lifespan shortening under the HFD and HSD regimes, respectively [28,31], while the fat accumulation does not seem to be causative of aging in these types of *Drosophila* obesity. 

### 4.6. The Gut

The gut plays a central role in the digestion and absorption of nutrients. In addition, the gut regulates energy homeostasis via its endocrine and neuroendocrine functions [166]. The epithelial cells of the midgut produce at least 12 different peptide hormones [166]. These peptides include, for example, regulators of feeding, such as neuropeptide F (NPF) [167] and CCHa2 [60,168], and regulators of lipid metabolism, such as Tachykinin (Tk) [169]. The digestive tract also express starvation-inducible gene *takeout* (*to*) [166], which codes for a juvenile hormone-binding protein that promotes feeding [170,171] and extends lifespan [172]. Interestingly, *to* is activated under several lifespan-extending manipulations, including dietary restriction, downregulation of the IIS pathway, *mth* mutation, and repression of ecdysteroid signaling [172,173], 

The adult gut of *Drosophila* is a plastic organ capable of regeneration; epithelium of the midgut renews within two weeks [166]. Maintaining intestinal homeostasis is important for healthy aging [174]. Senescence of the gut is associated with leakage of the epithelial barrier, increase in the proliferation of the stem cells and accumulation of the undifferentiated progenitor cells [166,174]. The loss of the tissue homeostasis leads to inflammation-like state, which activates the stress-induced Jun-N-terminal kinase (JNK) pathway [175]. The JNK pathway promotes further overproliferation of intestinal stem cells, which is directly responsible for lifespan shortening [176]. Overactivation of the JNK pathway also leads to the chronic activation of the IIS-repressible factor Foxo, which inhibits expression of the lipase Magro (Mag) [175], the key lipase necessary for digestion of triacylgylcerol [177]. Reduced Mag activity thus leads to lowered body fat content in old flies [175]. Experimental inhibition of the JNK pathway, or overexpression of Mag in the intestine increases fat reserves in old flies [175]. Moderate inhibition of the JNK pathway also extends lifespan [176], but whether this effect is mediated by improved lipid metabolism remains unclear. Mild increase in the intestinal Foxo signaling in aging flies might actually be an adaptive response, as lifespan is extended by overexpression of Foxo by GeneSwitch drivers with strong intestinal expression patterns, such as S106- and S32-GAL4 [154,178,179].

The microbiome of the fly gut depends on the diet [130], and contributes to the regulation of energy metabolism [180]. Elimination of the microbiota leads to obesity, despite lower food intake of axenic flies [180]. The effect of the gut bacteria on lifespan is less clear, as both lifespan shortening effect [181], as well as longevity where reported for axenic flies [182]. The effect of the antibiotic treatment on lifespan likely depends on the sex and age of the flies when antibiotics are applied [183]. 

Altogether, maintenance of the gut homeostasis is required for proper digestion and uptake of lipids, as well as for longevity. In addition, the gut may link energy metabolism and longevity via numerous endocrine and neuroendocrine factors, and via the gut microbiota.

## 5. The Adaptive Roles of Hyperglycemia and Obesity 

### 5.1. The Thrifty Genotype

Diabetes mellitus type 2 is a clearly harmful, nevertheless, surprisingly widespread disease. In 1962, James Neel proposed the “thrifty genotype hypothesis” [184], arguing that in the course of evolution, “thrifty” genetic variants that promoted accumulation of fat were positively selected, because they enabled survival during famine periods. However, these genotypes became maladaptive in modern societies where nutrition is not restricted, being now responsible for obesity, diabetes type 2, and other metabolic complications. The thrifty genotype hypothesis predicted the existence of a genetic predisposition for obesity and diabetes, and recent genome-wide association studies indeed identified numerous obesity-associated loci [185]. Like human societies in the past, natural populations of *Drosophila* are exposed to fluctuations in the food availability. Interestingly, there is also a genetic variation in the fly genes associated with fat accumulation [127,128,129,130], suggesting that the thrifty genotype hypothesis might explain the evolution of some types of *Drosophila* obesity as well.

### 5.2. Lipogenesis as a Protective Mechanism against Glucose Toxicity

Lipogenesis can be considered as a protective mechanism of sugar detoxification [25]. The evolution of fat storage not only enables survival during periods of food shortage, but also provides a protection against hyperglycemia. However, when lipogenesis exceeds the storage capacity of the adipose tissues, fat starts to accumulate in other organs. The ectopic fat storage elicits lipotoxicity, which, in humans, leads to damage of the pancreatic beta cells, cardiomyocytes, hepatocytes, renal parenchymal cells, and endothelial cells [186]. It has been suggested that many medical complications of obesity result from the lipotoxic response in non-adipose tissues, rather than from accumulation of fat in the adipocytes [186]. Thus, an increase in the lipid storage capacity of adipocytes might actually be beneficial. This notion has been confirmed in the *Drosophila* models of the HSD-induced obesity, in which an experimental increase in the fat storage improved tolerance to dietary sugars, whereas genetic interventions leading to a lean phenotype exacerbated the consequences of HSD [25]. These experiments highlight the importance of understanding the physiology of different types of obesity, as the reduction of body fat may actually worsen health under certain conditions.

### 5.3. The “Obesity Paradox” and the Beneficial Roles of Body Fat

The fat reserves clearly provide an advantage under conditions of nutrient scarcity. Nevertheless, numerous studies showed that the fat might have a protective role under ad libitum feeding as well. For example, increased BMI correlates with an improved prognosis and lowered mortality in several medical conditions. These include, for example, chronic heart failure and coronary diseases [187,188], end-stage kidney disease [189], and cancer [190]. This phenomenon—a better prognosis of obese individuals—is known also as the “obesity paradox”.

Obesity typically correlates with increased all-cause mortality [3,4]. Nevertheless, a meta-analysis study by Flegal et al. [2] showed that whereas this is true for obesity (BMI ≥ 30), overweight (BMI of 25 < 30) is actually associated with a significantly lower all-cause mortality, suggesting that there are instances in which increased body fat provides an advantage. A positive role of stored lipids has been shown in several others systems, including the budding yeast *Saccharomyces cerevisiae*, in which excessive fat increases lifespan [191]. A recent review on the protective roles of triacylglycerides argued that neutral fat prolongs lifespan in an energy-independent fashion, by protecting against various stressors [192]. Interestingly, lipid droplets have antioxidant roles and protect stem cells also in flies [193], suggesting that this beneficial function may involve other fly tissues as well. Longevity models of *Drosophila*—such as the flies with downregulated TOR [34] and IIS [32] signaling, and the *Methuselah* mutants [194]—have increased resistance to paraquat, a commonly used agent to elicit oxidative stress in insects. Thus, it is tempting to speculate on a potential antioxidant role of the lipid droplets in these long-lived obese flies. 

### 5.4. Adaptive Roles of the Stress-Induced Hyperglycemia

Chronic diabetes is dangerous for human health, yet there are instances when hyperglycemia provides an advantage. In contrast to the chronic hyperglycemia, stress hyperglycemia is an important, evolutionarily conserved mechanism to cope with injury and infection (reviewed in [195,196]). In humans, this type of hyperglycemia is hormonally regulated by the hypothalamus–pituitary–adrenal axis, sympathoadrenal system, and proinflammatory cytokines, which collectively induce gluconeogenesis, glycogenolysis, and insulin resistance [195]. Stress-induced hyperglycemia was reported in organisms ranging from worms to humans [196]. In *Drosophila*, an increase in circulating sugars seems to be a common response to various forms of infections, including bacterial [197], nematode [198], and wasp parasites [199]. This mechanism is highly adaptive, documented by the fact that inhibition of the rise in circulating sugars, either by inhibition of the adenosine transport or AKH signaling, increases sensitivity to the infections [198,199]. The infection-induced hyperglycemia is likely mediated by the attenuation of the IIS pathway, as shown, for example, for the infection by *Mycobacterium marinum* [197]. An experimental decrease of the IIS (by overexpression of *foxo* or by mutation in the InR substrate *Chico*) is sufficient to increase the transcription of several immune genes [200]. Thus, it seems that the increase in circulating sugars driven by the low IIS is sufficient to enhance *Drosophila* immunity. As a case in point, the glucose-based HSD increases glycemia and resistance to infection by the enteric pathogen *Vibrio cholerae* [21]. However, the sucrose-based HSD increases susceptibility to infection by *Pseudomonas aeruginosa* [68]. Thus, it remains to be determined whether these inconsistencies result from differential effects of dietary sugars (glucose [21] vs sucrose [68]), or rather reflect pathogen-specific effects of HSD. 

## 6. Limitations of the Comparisons among *Drosophila* Studies

Comparisons of individual studies on obese flies is limited by the variability in the methods used to analyze and express the fat content (fat per fly, or normalized per body weight or per protein content), as well as by the differences in the diets used across laboratories. There is no clear definition of fly obesity yet; nevertheless, the normalization of fat content to the protein levels could fulfill a similar function as the BMI index in humans. The field of *Drosophila* research would also undoubtedly benefit from standardized diets, and from reporting the raw lipid and protein data in all obesity studies, which would allow meaningful comparisons and meta-analyses of individual papers. 

## 7. Potential Roles of the Fat Body Dynamics in Longevity

Even though fat accumulation in *Drosophila* often correlates with longevity (e.g., [21,32,33,34,35,36,85]), experimental increase in the fat content via inhibition of the lipolytic pathways, for example, by mutations of *bmm* or *Akh*, moderately decreases lifespan ([134] and M.G. and P.K unpublished data [135]). Conversely, an increase in lipolytic AKH signaling extends lifespan [91,136], despite causing a lean phenotype [53,54]. Studies by Katewa and colleagues [90,91] argued that lifespan is extended by an increase in the fat turnover, i.e., by increase in both lipogenesis and lipolysis, irrespective of whether it is associated with a lean phenotype (as in the case of *Akh* overexpression [91]), or with obesity (as in the case of DR [90]). This hypothesis also explains the decrease in the lifespans of the *bmm* [134] and *Akh* mutants (M.G. and P.K., unpublished data [135]), as their defects in lipid mobilization probably reduce the global fat turnover rates. 

However, how the DR-induced turnover is facilitated at the cellular level remains an open question. Is it specific for the triacylglycerides, or does it reflect a general increase in the turnover of all macromolecules, or even organelles? The longevity effects of the IIS and TOR downregulations are associated with increased autophagy-mediated turnover of organelles [201,202,203]. Importantly, as shown in mice, autophagy has an important role in lipid metabolism, promoting lipolysis and breakdown of lipid droplets [204]. Thus, increased fat turnover might be linked to longevity via the well-known lifespan-extending effects of autophagy [203]. 

The relation between obesity, aging, and the dynamics of the fat body at the cellular level remains enigmatic as well. In contrast to the larval fat body that consists of post-mitotic cells that grow solely via endocycling [205], the cellular biology of the adult fat body is not well understood. The number of fat body cells remains rather constant throughout life, while their size increases several fold within the first days after eclosion [206]. The fat body cells are susceptible to stress-induced apoptosis [207], can adapt to the increased adiposity by increasing the cellular proportion of lipid droplets [48,49], and possibly also by growth and division [206]. Nevertheless, how these multinucleate and polyploid cells proliferate, is not known, and the existence of fat body stem cells has not been reported to date. The mechanism whereby the lifespan-extending manipulations affect the physiology of this tissue remains to be investigated. Nevertheless, differential contribution of fat turnover, autophagy, cell proliferation and growth in different types of obesity might explain why certain types of obesity are coupled with lifespan shortening, whereas others are associated with longevity. In conclusion, the so far unknown cellular biology of the *Drosophila* fat body may hold the key to a better understanding of the interactions between fat storage, inflammatory responses, and lifespan. 

## Figures and Tables

**Table 1:** Examples of obesity-causing manipulations and their effects on the lifespan of *Drosophila.* Legend: ↑ increase; ↓ decrease; - no change; nd not determined in the study.

Obesity Type		Details	Study	Fat	Glycemia	Lifespan	Note
Diet-induced	HSD	sucrose	[23]	↑	nd	↑	Increase in the mean lifespan, but also increase in the early mortality.
		glucose	[21]	↑	↑	↑	
		sucrose	[31]	↑	↑	↓	Inhibition of the hexosamine pathway rescues lifespan.
		sucrose	[19]	↑	↑	↓	
		sucrose	[71]	↑	nd	↓	Importance of the protein/carbohydrate ratio.
	HFD	lard	[28]	↑	↑	↓	Inhibition of the immune response rescues lifespan, but not obesity.
		coconut oil	[29]	↑	nd	↓	Obesity and decrease in lifespan are ameliorated by endurance exercise.
		coconut oil	[27]	↑	↑	↓	Increased levels of the total body glucose.
	dietary restriction	amino acid restriction	[35]	↑	-	↑	
		yeast restriction	[91]	↑	nd	↑	
Genetic	IIS related (reduced IIS)	*Ilp2-3*,*5* mutants	[33]	↑	nd	-	
		*Ilp2 > rpr*	[32]	↑	↑	↑	Ablation of the insulin-producing cells in the brain.
		*hsGAL4 > ImpL2*	[65]	↑	-	↑	Heat shock-inducible overexpression of *ImpL2.*
		*S106 > Ilp6*	[138]	↑	↑	↑	Overexpression of *Ilp6* in the fat body.
		*Mth* mutants	[120]	↑	nd	↑	Increased lipid reserves inferred from the starvation resistance
	other	*female sterile (2) adipose* mutants	[103]	↑	nd	↓	Lifespan decreased in mated females.
		*bmm* mutant	[134]	↑	nd	↓	
		rapamycin	[34]	↑	nd	↑	Feeding with rapamycin (inhibitor of TOR).

## Abbreviations

*Akh*	*Adipokinetic hormone*
BMI	body mass index
*bmm*	*brummer*
*CCHa2*	*CCHamide-2*
DR	dietary restriction
*EcR*	*Ecdysone receptor*
*fit*	*female-specific independent of transformer*
*foxo*	*Forkhead Box, Sub-group O*
HFD	high-fat diet
*hh*	*hedgehog*
HSD	high-sugar diet
*IGF*	*Insulin-like growth factor*
*Ilp1–8*	*Insulin-like peptide 1–8*
IIS	insulin/insulin-like growth factor signaling
IL-6	interleukin 6
*ImpL2*	*Ecdysone-inducible L2*
*InR*	*Insulin-like receptor*
JNK	Jun-N-terminal kinase pathway
*mag*	*magro*
MHO	metabolically healthy obesity
*mth*	*methuselah*
*NPF*	*neuropeptide F*
*Tk*	*Tachykinin*
TNF-α	tumor necrosis factor-α
*to*	*takeout*
*TOR*	*target of rapamycin*
*upd2*	*unpaired 2*
*upd3*	*unpaired 3*
